# FlexOracle: predicting flexible hinges by identification of stable domains

**DOI:** 10.1186/1471-2105-8-215

**Published:** 2007-06-22

**Authors:** Samuel C Flores, Mark B Gerstein

**Affiliations:** 1 Department of Physics, Yale University, Bass 432, 266 Whitney Ave. New Haven, CT 06520, USA; 2 Department of Molecular Biophysics and Biochemistry, Yale University, Bass 432, 266 Whitney Ave. New Haven, CT 06520, USA; 3 Department of Computer Science, Yale University, Bass 432, 266 Whitney Ave. New Haven, CT 06520, USA; 4 Computational Biology and Bioinformatics Program, Yale University, Bass 432, 266 Whitney Ave. New Haven, CT 06520, USA

## Abstract

**Background:**

Protein motions play an essential role in catalysis and protein-ligand interactions, but are difficult to observe directly. A substantial fraction of protein motions involve hinge bending. For these proteins, the accurate identification of flexible hinges connecting rigid domains would provide significant insight into motion. Programs such as GNM and FIRST have made global flexibility predictions available at low computational cost, but are not designed specifically for finding hinge points.

**Results:**

Here we present the novel FlexOracle hinge prediction approach based on the ideas that energetic interactions are stronger *within *structural domains than *between *them, and that fragments generated by cleaving the protein at the hinge site are independently stable. We implement this as a tool within the Database of Macromolecular Motions, MolMovDB.org. For a given structure, we generate pairs of fragments based on scanning all possible cleavage points on the protein chain, compute the energy of the fragments compared with the undivided protein, and predict hinges where this quantity is minimal. We present three specific implementations of this approach. In the first, we consider only pairs of fragments generated by cutting at a *single *location on the protein chain and then use a standard molecular mechanics force field to calculate the enthalpies of the two fragments. In the second, we generate fragments in the same way but instead compute their free energies using a knowledge based force field. In the third, we generate fragment pairs by cutting at *two *points on the protein chain and then calculate their free energies.

**Conclusion:**

Quantitative results demonstrate our method's ability to predict known hinges from the Database of Macromolecular Motions.

## Background

Proteins fold reliably into conformations essential for their function. The coordinates reported as representing a protein structure, however, are in fact averages over an ensemble at low temperature, at least when solved by X-ray crystallography. Specific motions are thermodynamically permitted about this equilibrium position and often play an important role in enzyme catalysis and protein-ligand interactions. The motions can be classified according to the size of the mobile units, which may be fragments, domains or subunits[1,2] They can be further classified on the basis of packing as shear, hinge, or other [1-3].

The mechanism of motion is difficult to observe directly. NMR studies can yield root mean square fluctuations and order parameters[4]. Optical trapping [5] can be used to track the movement of molecular motors. Hydrogen/deuterium exchange can be used to measure changes in the solvent exposure of amide protons[6]. The hinge connecting two independently folded domains in a protein is sometimes a sensitive site for proteolytic cleavage[7]. Many of these experimental techniques, however, require much effort and provide limited information[8].

Computational simulations have been used for several decades to predict protein dynamics. However expense generally prohibits the all-atoms modeling of large systems without substantial simplifications[9]. Even for systems of moderate size, hinge bending and other large scale backbone rearrangements often take place on time scales inaccessible to Molecular Dynamics. Normal mode studies can be performed using the simplified GNM treatment, but often multiple modes are necessary to represent the motion[10], and it is not necessarily clear a priori which modes are important. Yang et al[11], for example, show that squared-displacement minima of the first *two *nontrivial modes are correlated with active site location, and argue that this is the hinge point. Similarly, Rader et al[12] showed that fluctuation minima of the one or two slowest modes avoid the folding cores of proteins, and argued that these coincide with interdomain hinges. Kundu et al[13] use the lowest order nontrivial mode to assign residues to one of two structural domains according to the *sign *of the displacement, and also perform some physically motivated postprocessing of the results.

Similarly, much work has been done to solve the related problem of finding domain boundaries, which can be flexible or inflexible. Nagarajan and Yona[14] have shown how to analyze multiple sequence alignments to identify domains. Marsden et al showed that predicted secondary structure could help find domain boundaries. Jones et al. combined PUU[15], DETECTIVE[16], and DOMAK[17] to make a powerful domain boundary predictor[18]. Domain boundaries, again, are not necessarily flexible, and furthermore many of these methods require a multiple sequence alignment which cannot always be obtained. Given the difficulty of observing motion by experimental means and the limited accuracy or applicability of existing computational methods, there is a need for improved techniques for predicting motion.

45% of motions in a representative set from the Database of Macromolecular Motions have been found to move by a hinge bending mechanism [1-3]. Keating et al.(in preparation) found that interpretation of hydrogen-bond dilution plots produced by FIRST[19] could discriminate domain hinge bending from fragment motions with some accuracy, even when the motion itself was unknown. For hinge bending proteins, if the location of the hinge could be predicted given a single set of structural coordinates, significant insight could be gained into possible movements.

Numerous valuable contributions have been made to the computational prediction of protein hinges. If the structure has been solved in two different conformations, then the hinge can be identified by visual inspection (Flores et al., submitted) or by use of FlexProt[20] or DynDom[21]. A much more difficult problem arises when only *one *conformation is known. In an early contribution, Janin and Wodak[22] developed a domain interface area calculation method. The FIRST algorithm[19,23-26] uses graph theory to economically identify rigid substructures. FRODA uses geometric simulation under constraints assigned by FIRST to generate alternate conformations of proteins which have been shown to be consistent with crystallographic and NMR data for certain proteins[9], but this ignores many important intra-molecular interactions and is more useful for loop motions than for domain hinge bending. Similarly, DisEMBL[27] successfully predicts flexible or disordered regions in proteins using a neural network, but this local flexibility alone is not a very strong predictor of hinges (Flores et al., submitted). The TLSMD[28] procedure analyzes the distribution of atomic displacement parameters associated with the mean position of each atom, and generates Translation-Libration-Screw descriptions of rigid groups of atoms, but has no means of identifying the group responsible for the principal hinge bending mechanism, and is limited to X-ray crystal structures of sufficient resolution. The Gaussian Network Model (GNM)[29] is an approximate algorithm for normal mode extraction widely used in flexibility prediction. FlexOracle is a complementary new addition to this set of tools.

## Methods

Domains can move relative to each other only if the motion is permitted energetically. Thus if two domains have many interdomain interactions they are unlikely to separate. Similarly, if a motion results in the exposure of large hydrophobic areas on the protein, then the energetic and entropic cost of solvation will make that motion less likely to occur.

For these reasons, we argue that if two or more domains are joined by a hinge, and if a peptide bond is broken on the protein, the energetic cost of separating and solvating the two resulting fragments will be lowest if that break is in a hinge. Conversely, if the break is inside a rigid domain, the energetic cost will be high. We will show how this idea leads to a hinge prediction method.

### Single-cut hinge predictor (TINKER version)

The idea of evaluating the cost of separating two fragments can be implemented using the minimization and single point energy evaluation features available in almost any molecular mechanics engine. This energy of separation is equivalent, up to an additive constant, to the difference in enthalpies between the two fragments generated by introducing a single cut on the protein chain on the one hand, and the original, undivided chain on the other hand. This energy evaluation can be carried out for every choice of cut location, and the resulting energy vs. cut location graph should have minima at locations that coincide with flexible hinges between domains. We will explain the methodology in detail.

**Figure 1:**
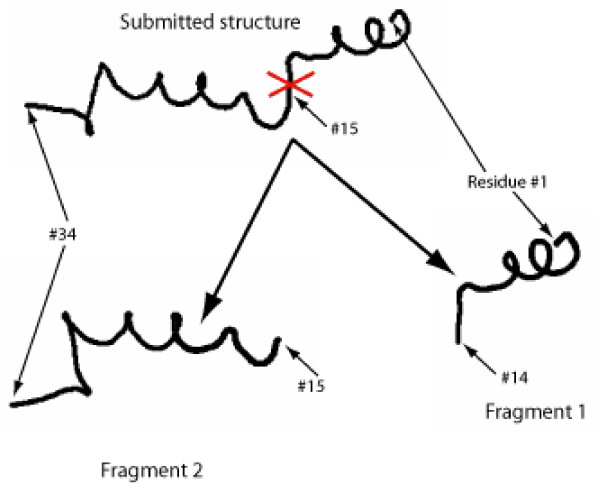
A key step in the FlexOracle method: separating the protein into two fragments, as illustrated here for i = 15.

We start with an *energy minimization step*, to relieve any close contacts or unnatural bond lengths or angles in the undivided chain which would bias the results. For this we use TINKER's *minimize *routine with the OPLS-All Atom[30] force field and the Ooi-Scheraga Solvent Accessible Surface Area (SASA)[31] continuum solvation free energy term. For each iteration of the predictor, we introduce a cut between *residues i *– *1 *and *i*. This divides the protein into two fragments, numbered 1 and 2 (Figure 1). Fragment 1 is a polypeptide containing residues *1 *to *i *– *1*, and fragment 2 is another polypeptide containing residues *i *to *N*. We use these fragments in an energetic calculation as follows. We define *E*_*C *_as the single point energy of the complete (undivided) protein. This includes bonded and non-bonded interactions. In the *energy evaluation step *we again use the OPLS-All Atom force field with the SASA implicit solvent model. Note that this step, and this step alone, will change in the second variant of FlexOracle.

For each choice of cut location *i*, we compute fragment single point energies *E*_*frag*1_(*i*) and *E*_*frag*2_(*i*). We argue that Δ*E*(*i*) = *E*_*frag*1_(*i*) + *E*_*frag*2_(*i*) - *E*_*C *_is related to the energy change associated with hinge motion about the selected hinge, as follows.

The quantity Δ*E*(*i*) represents the *intra*-fragment energy gained or lost by breaking all of the interactions between fragment 1 and fragment 2, as might occur in an opening motion. It also includes the solvation energy which might be gained or lost. The quantity *E*_*C *_is a constant independent of the cut location and can be set to zero without consequence.

Even when the actual motion of the protein is not an opening one, the method should have predictive value because for *incorrect *choices of the hinge location, i.e. cut locations that are actually inside one of the domains, many *inter*-fragment interactions would be broken. Also, significant hydrophobic areas would be exposed on the surfaces of fragments 1 and 2. In either case, Δ*E*(*i*) would be relatively high.

Clearly, we can repeat the procedure of cutting the protein before residue *i *and computing Δ*E*(*i*) for values of *i *that are scanned from 2 through N. We then plot Δ*E*(*i*) vs. *i *and expect that minima on this graph will correspond to hinge locations.

It is to be expected that there exists a "single-cut" error associated with the fact that we are cutting the backbone at only one location. In many proteins, the backbone crosses the hinge region two or more times. Thus the single-cut predictor gives significantly clearer results for single-stranded hinges (e.g. Lir-1, see *Discussion of specific proteins*) than for double, triple, etc. stranded hinges (e.g. GluR2). We will return to this point later.

#### Identification of local minima

As will be discussed later for specific proteins, the *local minima *tend to coincide with hinges; globally lowest energy values were not the best indicators of flexibility. However many minima were generated by short range fluctuations in the predictor results which did not correspond to hinges. Therefore in order to clearly define which minima are most likely to correspond to hinges we used a moving window minimum identifier as follows.

First, the energies were normalized to range from 0 to 1. A given residue was considered to be a minimum if it had the lowest energy of any residue in a window that also included 8 residues to the left and right (for a total of 17 residues in the window). However it also had to be lower in energy than the *highest *energy residue in the window by 0.12. Lastly, residues less than 20 amino acids from either terminus were not considered as possible minima. Whenever any residue *i *was found to be a minimum, residue *i *- 1 was also considered to be a minimum. This is because as indicated earlier the energy value associated with residue *i *actually corresponds to a cut *between *residues *i *- 1 and *i*.

### Single-cut predictor (FoldX version)

Standard molecular mechanics force fields do not account for the backbone and side chain entropy, which is not needed to calculate dynamics. For our purposes entropy is important, since it is possible that changes in freedom of motion influence conformational change. Therefore we sought to improve the method by using the FoldX[32,33] force field.  FoldX includes terms that estimate the entropic cost of constraining the backbone and side chains in particular conformations. The interaction with solvent is treated mostly implicitly, although persistent entrained water molecules are treated explicitly. Other terms account for Van der Waals, hydrogen bonding, electrostatic, and steric interactions.

In the FoldX version of the single-cut predictor, the *energy minimization step *described above (for the TINKER version) was still carried out using the OPLS-All Atom force field, but in the *energy evaluation step*, also described above, calculation of the fragment energy was now carried out using the FoldX force field. All other steps were carried out exactly as for the TINKER version.

### Two-cut hinge predictor

Although accounting for the entropy was an important improvement, the method described above is still implicitly geared towards the detection of single-stranded hinges since it cuts the chain at a single location. One obvious way to deal with double stranded hinges is to make not one but *two *cuts in the backbone, at residues *i *and *j*. To do this the single index *i *was replaced with the indices *i *and *j*. These define two fragments consisting of the following residues:

Fragment 1: residues 1 to (*i *- 1) and (*j *to N)

Fragment 2: residues *i *to (*j *- 1)

We initially tried using CHARMm with the Born Solvation Model to compute the enthalpies of the fragments, but the computational expense was prohibitively high and the accuracy relatively low. We found that if instead we computed the free energy using FoldX, the predictor became accurate and the expense reasonable.

In order to find the choice of *i *and *j *corresponding to the hinge location one should ideally generate two fragments for every possible choice of *i*, *j *but in practice we found that restricting *i *and *j *to multiples of four was sufficient to locate the hinge in most cases and the resulting 16-fold reduction in computational expense brought the method into the realm of practical calculation on a single processor. Additional savings were obtained by restricting the range of *i*, *j*, to no fewer than 5 residues from either terminus and requiring that *i *≤ *(j-8)*, although numbers greater than 8 could potentially be used for even greater savings. To put this more concisely the calculation scheme looks like this:

for (*i *= 8 to N - 5 - 8 step 4)

   for (*j = i *+ 8 to N - 5 step 4)

      compute FoldX_energy (stability of fragment 1 + fragment 2)

**Figure 2:**
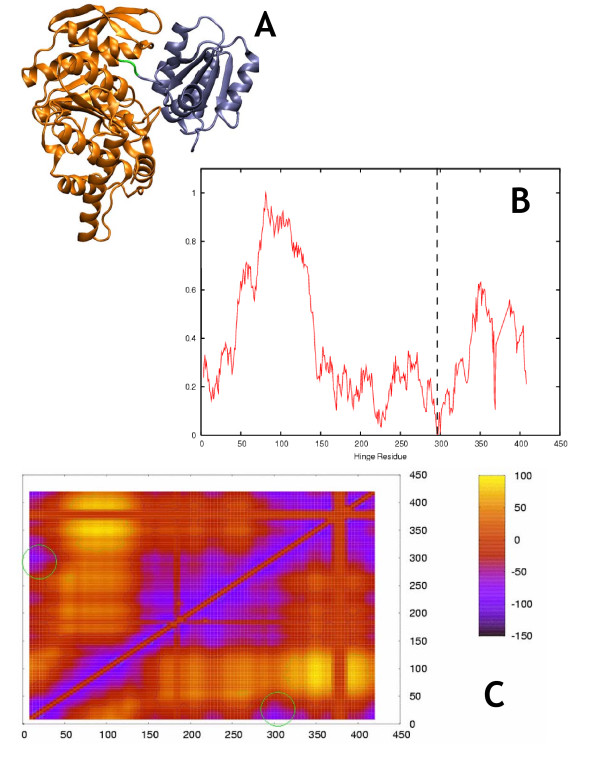
**Folylpolyglutamate Synthetase (closed)**. Morph ID: f046922-8341 PDB ID: 1jbw. Hinge Atlas Gold hinge: residues 296–297. a. Structure of FPGS, rendered by VMD in "New Cartoon" style through molmovdb's Render Studio. In this and all structural images in this work, coloring of the domains goes by the following logic. All the residues prior to the first hinge point are assigned to domain D1, all the residues between the first and second hinge points belong to D3, all the residues between the second and third hinge points belong to D1, and all subsequent residues belong to D3. The hinge residues themselves belong to D2. D1 is colored orange, D2 is green, and D3 is blue. Thus e.g. residue 1 is at the orange terminus, residues 295 and 296 are at the orange-green boundary, and no labeling is needed. b. Both versions of the single-cut predictor have clear minima on the energy plot near the correct hinge location at residue 297. GNM results were less successful. c. Graph key. For this and all FlexOracle graphs in this work, the dotted red line is the single-cut TINKER output, the solid red line is the single-cut FoldX output, and the dotted black line is the GNM first normal mode displacement. All three are normalized to range from 0 to 1. The green x's indicate the annotated hinge location from HAG. d. 2-cut FlexOracle makes a primary prediction at residues 298–301. This method was successful, since the first prediction was close to the HAG hinge, circled in green.

**Figure 3:**
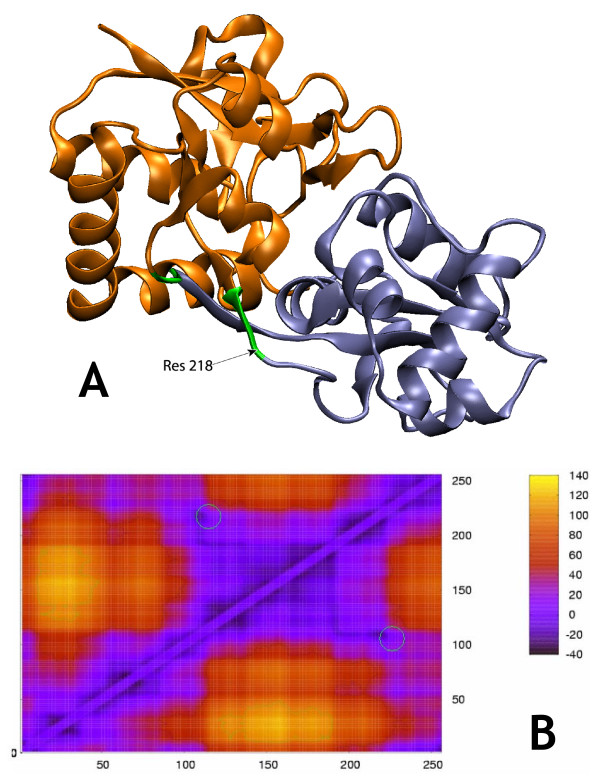
**AMPA Receptor GluR2 (closed)**. Morph ID: f437610-635 PDB ID: 1ftm. Hinge Atlas Gold hinges: 105–106, 218–219. b. The "mountain and shoulders" profile discussed in the text is clearly visible here. c. 2-cut FlexOracle primary hinge prediction: residues 108–111 and 216–219. Prediction was successful. Green circle indicates HAG hinge position.

**Figure 4:**
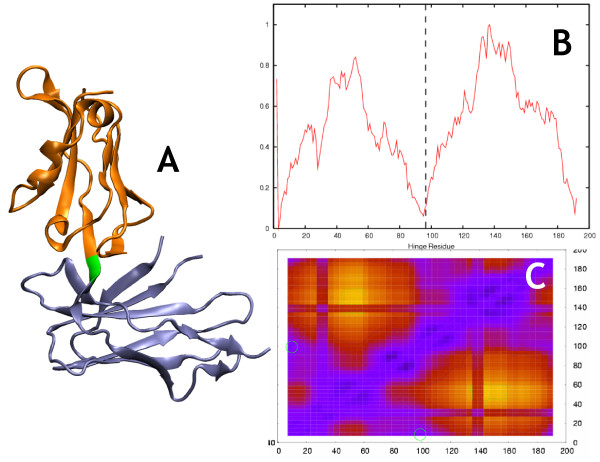
**Lir-1 (closed)**. Morph ID: f263558-23071 PDB ID: 1g0x. Hinge Atlas Gold hinge: 96–97. a. The HAG hinge is close to the proteolytic cleavage site between residues 99 and 100 as described in the text. b. The single-cut predictor results could hardly be less ambiguous, with both versions returning a clear minimum near the hinge location. c. 2-cut FlexOracle primary prediction: 97–100. The method was successful in this case.

**Figure 5:**
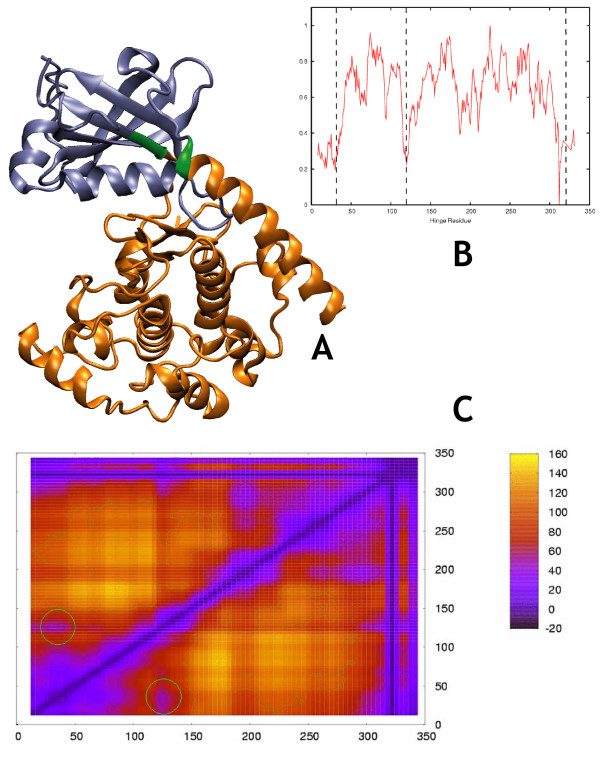
**cAMP-dependent protein kinase (open)**. Morph ID: f048180-370 PDB ID: 1ctp. Hinge Atlas Gold hinges: 31–32, 119–120, 319–320. b. Zheng et al. identify the boundaries of the small lobe as residues 40 and 127, slightly different from HAG. The single-cut predictors had significant minima near residue 120, with more ambiguous results for the other two hinges. c. 2-cut FlexOracle primary prediction: residues 314–317. Others: 30–33, 62–65. 42–45, 82–85. 122–125. The 2-cut predictor was partially successful. The primary prediction coincides with one of the hinges, as does the fourth prediction, and one of the second predictions. There are also three false positives (62–65 and 42–45, and 82–85) among the higher predictions.

**Figure 6:**
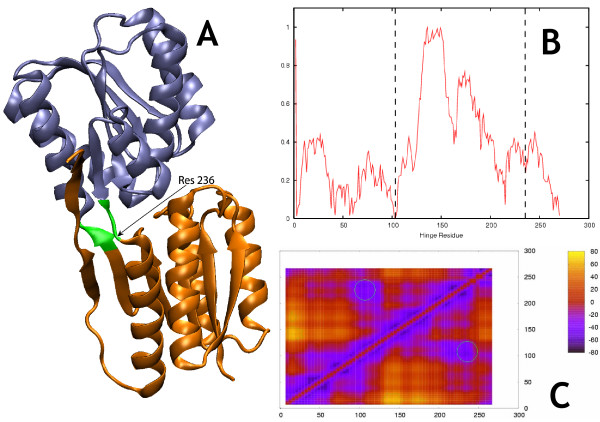
**Ribose binding protein (open)**. Morph ID: f924994-9791 PDB ID: 1drj. Hinge Atlas Gold hinges: 103,104,235,236. b. The single-cut predictors correctly suggest the hinge at residue 103, but less clearly at residue 235. Several false positives can also be seen, at residue 135 and around residue 50. c. The 2-cut predictor yielded the correct result, as indicated by the minimum circled in green.

**Figure 7:**
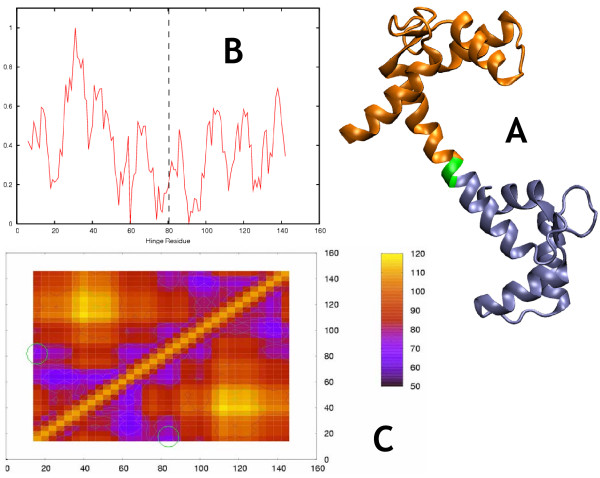
**Calmodulin (open, calcium bound form)**. Morph ID: f958972-2168 PDB ID: 3cln. HAG hinges: 80,81. b. The TINKER version of the single-cut predictor gives ambiguous results for this calcium-bound protein, but good results for the calcium-free form [37]. The FoldX single-cut predictor, worked well for both calcium-bound and calcium-free forms Calmodulin. Nonetheless we recommend caution when treating metal-bound proteins, since the two-cut predictor had mixed results here. c. The 2-cut predictor results: Primary prediction: residues 30–33, 66–69. Additional predictions: 104–107;84–87. Although the primary prediction does not coincide with the annotated hinge, upon inspecting the corresponding morph (78252-5656) we observed that indeed there are hinges coinciding with the predicted location, although they result in less backbone motion than the hinge at residue 80. Similar points (residues ~36,63) are annotated hinges in the evolutionarily related Troponin C (morph 333010-30921). In the interest of maintaining the objectivity of the HAG, we did not update the hinge annotation. We further note that the third lowest-energy local minimum (84–87) is close to the HAG hinge. Thus although the first prediction did not coincide with the HAG, the results nonetheless yield significant insight into the flexibility of the protein.

The free energy of folding for each of the two fragments was computed separately by means of a 'Stability' run in FoldX 2.5.2. FoldX_energy is the sum of the two energies. Once FoldX_energy was calculated for all such pairs of fragments it was plotted, with energies coded with blue = lowest energy and yellow = highest as shown in figures 2, 3, 4, 5, 6, 7. Upon inspecting these graphs and comparing local minima of free energy to the known hinge locations, we found that the following cases occurred:

**Figure 8:**
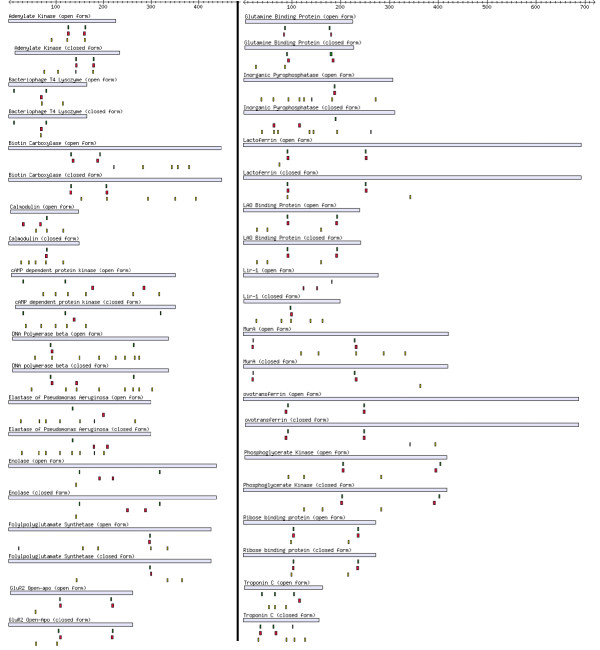
Comparison of two-cut FlexOracle hinge predictions (red bars) vs. HAG hinges (green bars). Light blue bars represent protein sequence. Residue numbers are given by the scale at the top of the figure. Labels give the structure ID for viewing on our server, the name of the protein, the conformation (open or closed), and the outcome (success, partial success, failure) according to the loose criterion described in the text. There is a clear tendency for FlexOracle predictions to align with the annotated hinge location and to correctly discriminate between single and double stranded hinges. The predictor was clearly less successful with triple stranded hinges (cAMP dependent protein kinase, Troponin C).

1. The *i*, *j *indices of a minimum were near the diagonal, meaning the corresponding fragment 2 was small. Such minima were discarded since the diagonal energies are generally small and we are not interested in small fragment motions.

2. Both *i *and *j *were near the termini. These minima were also discarded, because the termini are usually flexible but we are not studying those motions.

3. Of the minima that did not fall in cases 1 or 2, the lowest minimum sometimes had one of its two indices near a terminus, but the other substantially far from either terminus. In this case the former index was discarded for the reasons cited in (2) but the latter index tended to coincide with a single-stranded hinge.

4. Of the minima that did not fall in cases 1, 2, or 3, the lowest very often indicated the location of a double stranded hinge.

5. Lastly, on occasion the minimum reported following cases (3) or (4) did not correspond to the known hinge location, but one of the higher minima not eliminated per cases 1 and 2, did.

To identify and deal with the various cases, some clustering and postprocessing were needed, as follows.

#### Culling

As a preliminary step, we flagged all choices of *i, j *that resulted in

FoldX_energy < min(FoldX_energy) + (max(FoldX_energy) - min(FoldX_energy))·0.1

If this resulted in fewer than 30 fragment pairs, we instead flagged the 15% of pairs with lowest energy. All the remaining (unflagged) elements were not considered to be candidates for the hinge location.

#### Clustering

The next step was to identify and separate the local minima, for which we employed the k-means clustering algorithm. Centroids were initially generated in a regular grid spaced 50 residues apart starting at *i*, *j *= 25, 25. The pairs flagged in the culling step were each assigned to the nearest centroid. The location of each centroid was then recomputed for each resulting cluster, and the pairs were once again reassigned to the nearest recomputed centroid. This process was repeated until all centroids stopped moving. The lowest-energy element of each cluster was taken as the *local *minimum corresponding to that cluster.

#### Determination of hinge location

The minima found in the preceding step were recorded in order of energy, with the lowest corresponding to the *global *minimum. Any minima such that *i *≥ *(j - 24) *were discarded since they border the diagonal, per case (1) above. If for any minimum both *i *and *j *were within 20 residues of the termini, that minimum was also discarded, per case (2). For the lowest remaining minimum, if only *one *of the two indices was within 20 residues of a terminus, then the protein was identified as having a single-stranded hinge, per case (3). The index near the terminus was discarded and the remaining index was taken to be the location of the single-stranded hinge. Otherwise, both indices were taken together to indicate the location of a double stranded hinge, per case (4). Since the calculation was done only for every fourth residue, the hinge prediction was reported as a range:

Hinge 1: residues *i *-2 to *i *+1

Hinge 2: residues *j *-2 to *j *+1

Case (5) occurred somewhat less frequently, and so although our program outputs the remaining local minima these are much less accurate than the primary hinge prediction and were not used in the *Statistical evaluation *section. We do, however, discuss these secondary predictions in the *Discussion of specific proteins *section.

## Results

We tested our method against 20 pairs of protein structures (40 total structures), in the Hinge Atlas Gold (HAG), a dataset of manually annotated hinges publicly available on our Database of Macromolecular Motions[2,3,34-36]. We present the results in a summary statistical form and also discuss the individual results for six structures of the 40.

The HAG provides a carefully curated collection of 20 homologous pairs of single-chain protein structures[37]. Its name reflects its origin in the Hinge Atlas, a much larger set of morphs with annotated hinge locations. The latter is not suited for our purposes since it contains structures stabilized by large ligands, subunits of large complexes, and other cases requiring special treatment. The HAG is specifically compiled for the purpose of testing structure-based predictors of domain hinges and therefore includes only structures that meet the following conditions:

1. The structure is soluble and independently stable, rather than relying on other chains or molecules to maintain its conformation.

2. The structural coordinates were obtained by x-ray crystallography, with the exception of calcium-free calmodulin.

3. At least two sets of atomic coordinates are available, and together they represent a domain motion that is biologically relevant or thermodynamically feasible.

4. The motion involves two or more rigid domains moving about a flexible hinge.

Each of these pairs of protein structures, also known as morphs, has an annotated hinge location. This location was chosen prior to running any hinge prediction codes, by visual inspection of the corresponding morph movie. We have found manual annotation to be more reliable than the use of automated methods such as FlexProt, DynDom, or Hingefind, which depend on user-adjustable parameters and sometimes incorrectly assign the hinge location. The process of inspection and annotation was aided by the "Hinge Annotation Tool" available on the morph page for each morph in MolMovDB. It consists of a set of arrow buttons which adjust the position of a window of residues, which are highlighted as the protein moves. This tool can also take annotations from the public for various uses. The result of the annotation effort is a set of hinge residues for structural pairs against which FlexOracle and other hinge predictors can be tested.

One must bear in mind that the hinge annotation is not encyclopedic. It is based on the comparison of two sets of structural coordinates, but other motions not reflected by this measure may be thermodynamically feasible. In some cases FlexOracle predicted hinges not annotated in HAG but for which we later found experimental evidence in the published literature. Since the point of the HAG is to be objective rather than comprehensive, in these cases we did not change the annotation or our scoring of the predictor results. Some of these discrepancies are discussed in the *Discussion of specific proteins *section. First, however, we evaluate the performance of FlexOracle on the HAG as a whole.

### Statistical evaluation

As mentioned in the *Methods *section, FlexOracle assumes hinges do not simply correspond to points of *globally *lowest energy, but rather to *local minima *identified and postprocessed in various ways. The set of residues reported as predicted hinge locations by any of the three versions of FlexOracle are referred to as *test positives*, and the number of residues in this set we will call *M*. the residues annotated as hinges in the HAG are referred to as *gold standard positives*, and the number of these we will call *H*. In this section we compare the test positives to the gold standard positives to objectively evaluate the predictor. Before we do do so, however, we need to define a few more standard statistical terms as they relate to the current context:

*Gold standard negatives*: The residues in HAG that are NOT annotated as hinges.

*TP (true positives)*: The number of residues that were both test positives and gold standard positives.

*FP (false positives)*: The number of residues which were test positives and gold standard negatives.

*TN (true negatives)*: Number of residues which were test negatives and gold standard negatives.

*FN (false negatives)*: Number of residues which were test negatives and gold standard positives.

*Population*: All of the residues in the HAG. We will call the number of these residues *D. Sensitivity (true positive rate) *= TP/(TP + FN) = TP/H. This is the ratio of true positives to gold standard positives.

*Specificity (true negative rate) *= TN/(TN + FP) = TN/(D - H). This is the ratio of true negatives to gold standard negatives.

*Null hypothesis*: The statistical hypothesis that the set of *test positives *is not different from the *population *in a statistically significant fashion.

*Alternate hypothesis*: The hypothesis that the set of *test positives *is different from the *population *in a statistically significant fashion.

*p-value*: This is the probability that a set of residues numbering as many residues as are in the test positive set, and selected randomly from the *population*, would contain *TP or more *gold standard positive residues. If the p-value is above 0.05 we conventionally *accept *the *null hypothesis*, otherwise we *reject *the *null hypothesis *in favor of the *alternate hypothesis*. Clearly, the smaller the p-value the better the predictor.

The p-value is computed for all predictors in this study using the *cumulative *hypergeometric function,

p-value=∑x=TPMHYP(H,D,x,M)
 MathType@MTEF@5@5@+=feaafiart1ev1aaatCvAUfKttLearuWrP9MDH5MBPbIqV92AaeXatLxBI9gBaebbnrfifHhDYfgasaacH8akY=wiFfYdH8Gipec8Eeeu0xXdbba9frFj0=OqFfea0dXdd9vqai=hGuQ8kuc9pgc9s8qqaq=dirpe0xb9q8qiLsFr0=vr0=vr0dc8meaabaqaciaacaGaaeqabaqabeGadaaakeaacqqGWbaCcqqGTaqlcqqG2bGDcqqGHbqycqqGSbaBcqqG1bqDcqqGLbqzcqGH9aqpdaaeWbqaaiabdIeaijabdMfazjabdcfaqjabcIcaOiabdIeaijabcYcaSiabdseaejabcYcaSiabdIha4jabcYcaSiabd2eanjabcMcaPaWcbaGaemiEaGNaeyypa0JaemivaqLaemiuaafabaGaemyta0eaniabggHiLdaaaa@4BAA@

where the *hypergeometric function*[38] gives the probability of finding exactly *x *of the *H *gold standard positive residues in a set of *M *residues randomly chosen from the population numbering *D*:

HYP(H,D,x,M)=C(Mx)C(D−MH−x)C(DH).
 MathType@MTEF@5@5@+=feaafiart1ev1aaatCvAUfKttLearuWrP9MDH5MBPbIqV92AaeXatLxBI9gBaebbnrfifHhDYfgasaacH8akY=wiFfYdH8Gipec8Eeeu0xXdbba9frFj0=OqFfea0dXdd9vqai=hGuQ8kuc9pgc9s8qqaq=dirpe0xb9q8qiLsFr0=vr0=vr0dc8meaabaqaciaacaGaaeqabaqabeGadaaakeaacqWGibascqWGzbqwcqWGqbaucqGGOaakcqWGibascqGGSaalcqWGebarcqGGSaalcqWG4baEcqGGSaalcqWGnbqtcqGGPaqkcqGH9aqpdaWcaaqaaiabdoeadnaabmaabaqbaeqabiqaaaqaaiabd2eanbqaaiabdIha4baaaiaawIcacaGLPaaacqWGdbWqdaqadaqaauaabeqaceaaaeaacqWGebarcqGHsislcqWGnbqtaeaacqWGibascqGHsislcqWG4baEaaaacaGLOaGaayzkaaaabaGaem4qam0aaeWaaeaafaqabeGabaaabaGaemiraqeabaGaemisaGeaaaGaayjkaiaawMcaaaaacqGGUaGlaaa@4E90@

We will use the sensitivity, specificity, and p-value in our statistical evaluation. p-value is a particularly useful quantity, since it compares directly to random picking. The three quantities will be used to evaluate the three versions of FlexOracle and compare to GNM[29], long a popular flexibility prediction algorithm.

#### Single-cut predictors and GNM

We begin our statistical evaluation with the TINKER and FoldX versions of the single-cut predictor. We take as our *test positives *those residues identified as local minima according to the algorithm described in the *Methods *section, then tabulate the various statistical quantities per the above definitions. GNM requires a slightly different treatment. To evaluate this predictor, we compute the absolute value of the first normal mode displacements and normalize this quantity to range from 0 to 1. The *nodes*, or points of zero displacement, are taken to correspond to the hinge location. Therefore we take all residues with normalized displacement smaller than 0.02 to be test positives. The results are shown in Table 1.

**Table 1:** Summary of predictor results

		GNM	Single-cut predictor (TINKER)	Single-cut predictor (FoldX)	Two-cut predictor
1	Total residues in HAG	13246	13246	13246	13246
2	Test positives	1279	923	292	268
3	Gold Standard positives	152	152	152	152
4	True positives TP (2 ∩ 3)	39	24	14	62
5	False Positives FP (2 – 4)	1240	899	278	206
6	False Negatives FN (3 – 4)	113	128	138	90
7	True Negatives TN (*1–2–6)*	11854	12195	12816	12888
8	Sensitivity (TP/(TP+FN))	0.26	.157	.092	.41
9	Specificity (TN/(TN+FP))	.9053	.93	.98	.98
10	p-value	8.4·10^-9^	1.3·10^-4^	6.7·10^-6^	3.5·10^-66^

"Test positives" for GNM were those residues with first normal mode displacement below 0.02. Recall that the displacements are normalized to range from 0 to 1. For the single-cut predictors, test positives were residues identified as defining a local minimum per the algorithm described in the text. Lastly, "test positives" for the two-cut predictor are those selected under the strict criterion (4-residue window) also described in the text.

We observed qualitatively (figures 2, 3, 4, 5, 6, 7) that the FoldX version of the single-cut predictor was significantly less noisy, and therefore had fewer minima than the TINKER version (240 residues for FoldX vs. 923 for TINKER). This led to a lower sensitivity for the FoldX version, but improved specificity and p-value. GNM is less specific than either of the single-cut predictors, but has better sensitivity and p-value. This underscores the need to improve the single-cut predictor and further motivates the development of the two-cut predictor.

#### Two-cut predictor

The two-cut predictor was run on the 40 proteins in HAG and the results were compared to the hinge annotation. Note that as explained earlier test positives are reported by the two-cut predictor in windows 4 residues wide due to the 4-residue grid spacing. We refer to this window width as the *strict criterion *and use it for our statistical benchmark. The results are shown in Table 1. Note that the p-value is 3.5·10^-66 ^– indicating very high predictive power.

This proves the statistical significance of the test but in practice for a given protein a prediction that is in some sense *close enough *to the correct hinge may for practical purposes be considered a true positive even if it does not coincide *exactly*. Therefore for a more operational benchmark we widened the definition of the test positives to include 5 residues to the left and right of the predicted hinge location, for a window width of 14 residues (*loose *criterion). When a gold standard positive residue was found within the 14-residue window, this was considered a true positive. The test was considered a *success *for a given protein if there were no false positives or false negatives under this criterion. The test was considered a *partial success *if there were one or more true positives but also one or more false positives and/or false negatives. Finally, the test was a considered a *failure *if there were no true positives for that protein. The results are shown in Table 2. As can be seen, the majority of the proteins were successes.

**Table 2:** Summary of two-cut predictor results under the loose criterion (14-residue window)

Test result	Number of proteins
Success	24
Partial success	5
Failure	11

Under this criterion there were 47 true positive hinge points. For these, the average distance between the center of the gold standard positive residues and the center of the test positive residues was 1.66 residues. For 29 out of the 47, the distance was 1 or 0 residues. Thus even under the loose criterion the predictions had a tendency to line up closely with the HAG hinges. This can be appreciated in Figure 8, where the test positives are aligned with the corresponding gold standard positives, and the test outcome is indicated.

Also in the same figure one can observe that the predictor did not work well for the two pairs of proteins with triple-stranded hinges.

One must keep in mind that as we mentioned earlier, the HAG annotations reflect hinges chosen under a very specific crystallographic criterion and are not encyclopedic. Therefore for some of these "failures" it is possible that the prediction is correctly suggesting a motion which is thermodynamically permitted but is not reflected in the pairs of structures used to generate the hinge annotations. We will discuss this for specific cases in the following section.

## Discussion

We chose six representative proteins from the 40 structures in the HAG for detailed discussion. These reflect some of the diversity of the set and illustrate the salient features of the algorithm. For each of these, we present structural images with the annotated hinges highlighted. We also present and discuss the results of running the three versions of FlexOracle on the structure. The FlexOracle results for all 40 HAG structures can be viewed online[37].

The single-cut version of FlexOracle naturally works best on single-stranded hinges. This condition is less common, and in fact most proteins in HAG have two strands in the hinge, and a couple even have three. We will show that the single-cut predictor nonetheless has predictive ability in these cases, although the two-cut predictor is much more accurate.

The two-cut predictor, in contrast, is specifically designed to handle double-stranded hinges. It is also designed to respond to single stranded hinges by discarding one cut of the pair as described earlier. We did not attempt to extend the method to explicitly treat the case of triple stranded hinges.

Under either scheme, only one chain is analyzed at a time, in the absence of ligands, bound metals, or additional subunits of a complex. We show that the method is robust under removal of small ligands from co-crystallized coordinate sets. The method obtained mixed results with Calmodulin (see discussion below) so we do not recommend only careful use with metal-bound proteins. Similarly, care should be taken with single subunits taken from complexes, since these have not been tested rigorously.

### Folylpolyglutamate synthetase (FPGS) (closed)

Folate is a vitamin essential for cell growth and replication, in its sole function mediating the transfer of one-carbon units[39,40]. Folate must be polyglutamated by FPGS or else it may efflux from the cell[41]. In the polyglutamylation mechanism, a free carboxylate group on the folate molecule is activated in an ATP-dependent manner to give an acyl phosphate intermediate; this is followed by an attack by L-glutamate. FPGS forms a complex first with MgATP, then a folate derivate, and then glutamate, in an ordered manner in which the substrates are added sequentially. In the structure analyzed here, FPGS is in ternary complex with the non-hydrolyzable ATP analog β,γ-methylene-ATP (AMPPCP) and 5,10-methylenetetrahydrofolate (mTHF). These ligands are removed from the protein prior to analysis. Since both ligands are small, however, the open[37] and closed (Figure 2) conformers both yield predictions of roughly the same accuracy when tested with the single-cut predictors. This is true also for the two-cut predictor, for which the prediction agreed almost exactly with the HAG hinge for both open and closed conformers. Thus the removal of small ligands from the structural coordinate set does not significantly affect accuracy, a point explored further in the discussion of cAPK.

### AMPA-Sensitive Glutamate Receptor GluR2 ligand binding core (closed)

Ionotropic glutamate receptors (iGluRs) are responsible for fast synaptic transmission between mammalian nerve cells. iGluRs are a class of transmembrane proteins that form glutamate-gated ion channels, including the AMPA receptors GluR1-4. The transmembrane gate of iGluRs opens briefly in response to glutamate released by a presynaptic cell.

The GluR2 ligand binding core has been crystallized in progressively more tightly closed conformations, in the order of ligand binding apo>DNQX>kainite>glutamate~AMPA. This progression follows the binding affinity (e.g. GluR2 binds glutamate with higher affinity than kainite, and is more closed when bound with the former) except that AMPA binds with ~20-fold higher affinity than glutamate but produces the same effect on the conformation of the ligand binding core. The degree of closure, in turn, appears to control the receptor activation, as measured in terms of either peak current or steady state current in presence of the desensitization blocker cyclothiazide. Thus glutamate and AMPA are full agonists and produce the same maximal domain closure and consequent activation, whereas kainite is a partial agonist and results in lesser activation[42].

The well-characterized progressively stronger binding of the four ligands mentioned provides potentially fertile ground for motion prediction and ligand binding studies. In Figure 3 we show FlexOracle's results for the AMPA-bound structure. Domain 2 is a contiguous domain, by which we mean that it spans a single stretch of residues (106–218 according to the HAG definition), as apposed to domain 1, which is composed of two stretches 1–105 and 219–261 and is therefore discontiguous in sequence. Thus all cuts made by the single-cut predictor inside domain 1 leave domain 2 intact in one of the two fragments and necessarily break up domain 1, On the other hand, cuts made inside domain 2 break up both domains. The single-cut predictor graph exhibits a broad, high "mountain" of energy between the hinge residues 98 and 229, reflecting the cost of fragmenting domain 2. On either side of this region are broad "shoulders" of low energy, reflecting only the cost of breaking up domain 1, which cannot be avoided in a single-cut scheme. A similar "mountain" and "shoulders" profile can also be seen, albeit less clearly, for ribose binding protein (Figure 6) and for GBP and LAO binding protein[37]. The actual hinges appear not on the clear edges of the mountain but rather a few residues inside it. This reflects the fact that cutting near residues 98 or 229 keeps both strands of the close parallel double stranded linker in the same fragment (fragment 2 or fragment 1, respectively) whereas cutting at the actual HAG hinge locations would break up the interactions between the strands. Note that this hinge shifting effect does not occur for ribose binding protein, since the two strands of the hinge are not closely spaced along their full length and are not parallel.

Under the loose criterion, the two-cut predictor was successful in predicting the hinge.

### Leukocyte immunoglobulin-like receptor 1 (LIR-1) (closed)

The LIR family is composed of eight human proteins sharing significant sequence identity with LIR-1. LIR proteins are believed to be inhibitory receptors, similar to killer inhibitory receptors (KIRs) on human NK cells. LIR and KIR proteins belong to the immunoglobulin superfamily (IgSF). The extracellular region of LIR-1 contains four IgSF domains. The structure examined here is a fragment containing domains D1 and D2. The single-cut predictor results are clearly successful (Figure 4), since this is a single stranded hinge. The result of the two-cut predictor is likewise quite unequivocal; the method correctly detects that it is a single-stranded hinge and reports its location.

### cAMP-dependent protein kinase (cAPK) (closed)

Protein kinases modify substrates by transferring a phosphate from a nucleotide (typically ATP) to a free hydroxyl on a Ser, Thr or Tyr residue. The open conformation of cAPK appears to be stable in the apo form, as well as in complex with a peptide inhibitor. The closed form is stable in complex with peptide inhibitor and ATP. ATP precedes the peptide in an apparently preferred binding order[43].

The closed form is analyzed in Figure 5. FlexOracle strips the ligands from the protein, therefore one might naively expect diminished accuracy for the closed (ligand bound) case. After all, ligands of sufficient size might stabilize one or another of the rigid domains, and this seems likely to be the case for the binary complex. However in the trinary complex the ligand interactions also stabilize the closed conformation with respect to the open. Therefore separating fragment 1 from fragment 2, assuming *i *is a hinge residue, can be expected to require less energy without ligand than with. This argues that removing ligands from the structure should increase accuracy over the alternative. In fact the single-cut predictions are roughly as accurate for the closed conformer as for the open[37]. The two-cut predictor did not work well for either the open or closed conformer of this protein. When one considers that results were also poor for Troponin C (see Figure 8) it is clear that the two-cut predictor is not very good at detecting triple-stranded hinges.

### Ribose binding protein (RBP) (open)

RBP belongs to a sizeable family of soluble gram-negative bacterial periplasmic binding proteins with diverse ligands and functions. They are abundant and bind their substrates with high affinity and specificity, and thus easily sequester nutrients appearing in sporadically in the environment[44] The open conformation is predominant in the uncomplexed form. Upon ligand binding, the two separated domains close down around the ligand by virtue of a 30° rotation in the hinge that connects them.

Results for the apo form are shown in Figure 6. The single-cut predictor had a strong minimum at residue 103, and a weak one at residue 235, corresponding to HAG hinges at those locations. It incorrectly suggests flexible points around residues 208 and 50. The two-cut predictor worked perfectly for both open and closed conformers.

### Calmodulin (CaM) (open, calcium-bound form)

CaM is a major calcium-binding protein, regulating enzymes in many tissues[45]. It is known to exist in numerous vertebrate and invertebrate animals as well as plants. In spite of the wide phylogenetic variety of these organisms, the amino acid sequence of CaM is very highly conserved, with only seven amino acid substitutions, all conservative[45]. Troponin C has 50% sequence identity with CaM[45] and the two share structural features relevant to hinge finding. In particular, both can unwind at the same point near residue 80, although for Troponin C the biological significance of this is unknown. The two bind Ca^2+^, at the C-terminal lobe, but only CaM binds Ca^2+ ^at the N-terminal lobe. Correspondingly, the C-terminal lobes in the two proteins are structurally very similar to each other, while the N-terminal lobes are very different[46]. Both the single cut and the two-cut predictors find the hinge of calcium-free CaM clearly and unambiguously, as can be seen online[37]. For the calcium-bound form, the single cut TINKER predictor is ambiguous while the single-cut FoldX predictor is successful. The two-cut predictor fails completely (Figure 7), thus the results are mixed. Bound metals often have a significant stabilizing effect where they appear in proteins, as they are usually coordinated with multiple points on the polypeptide, and their removal would be expected to destabilize the protein significantly. The results for this protein suggest that although FlexOracle's neglect of small bound molecules is of little consequence, the neglect of bound metals may have a negative effect on the accuracy of the method. This may reflect the fact that a single divalent metal ion may have many strong interactions with neighboring protein atoms, whereas a small organic ligand has weaker interactions distributed over a greater area. Accordingly, small ligands tend to have significant thermal fluctuations about an equilibrium position, while metals tend to bind and coordinate neighbors in a very stable and position-specific manner. We therefore recommend care be taken when using this method for predicting hinges in metal-bound structures, when those metals appear to heavily affect the structural and motional characteristics of the protein.

## Web interface

Users may submit PDB-formatted files through our Hinge Prediction page, linked to from the MolMovDB front page[34]. They will receive an email with instructions on how to view graphs similar to those shown in figures 2, 3, 4, 5, 6, 7. In brief, the morph page contains a 'Hinge Analysis' tab which in turn has a link to the FlexOracle results. Blue diamonds on the single-cut predictor graph indicate the minima of the single-cut FoldX free energy per the criterion used in this work. Hinges tend to coincide with minima of the single-cut FlexOracle energy graph, as is explained in the *Discussion of specific proteins *section At this time only the single-cut predictor is run automatically on all submissions, but users may contact the author to have the two-cut predictor run on any submitted protein. The user should bear in mind that results may be of limited accuracy for membrane proteins and proteins bound to complexes or large substrates. If metals strongly affect the stability and motion of the protein, as is the case for EF hands, this may also limit accuracy. Lastly, if the hinge seems sterically unreasonable the reader should consider the possibility that the hinge has three or more strands or the motion is not hingelike.

The results of running FlexOracle and other hinge prediction algorithms on the HAG can be seen on our website[37]. Links to the corresponding morph page and detailed predictor results are provided. A full explanation of how to interact with the morph page is given in prior work[47].

## Conclusion

The ability of FlexOracle to predict the hinge location for domain hinge bending proteins was demonstrated. We found that FlexOracle gives similar results for apo and ligand bound structures when the ligand is a small molecule or molecules. However mixed results for the calcium bound form of calmodulin suggest care should be exercised when applying the method to proteins with bound metals. We further found that hinges often coincide with minima of the single-cut FlexOracle energy, but in the case of two-domain proteins comprised of one contiguous and one discontiguous domain, the hinge can occur instead near the boundary between a broad "mountain" of high energy (corresponding to the contiguous domain) and wide "shoulders" of low energy (corresponding to the discontiguous domain). Further, if the linker consists of closely spaced parallel strands, the hinge tends to occur a few residues into the "mountain" side of this boundary. Aside from the matter of bound metals, these issues are not a concern for the two-cut predictor, which is significantly more accurate than the single-cut predictor. The former works well for single as well as double stranded hinges, but not for triple-stranded hinges. The FlexOracle method addresses directly the problem of locating the primary hinge for hinge bending proteins.
